# Social Distancing and Shopping Behaviour: The Role of Anxiety, Attention, and Awareness on Safety Preferences while Queuing during the COVID-19 Pandemic

**DOI:** 10.3390/ijerph20054589

**Published:** 2023-03-05

**Authors:** George Horne, Adrian Furnham

**Affiliations:** 1 School of Sport, Exercise and Health Sciences, Loughborough University, Loughborough LE11 3TU, UK; 2 Department of Leadership and Organizational Behaviour, BI Norwegian Business School, 0484 Oslo, Norway

**Keywords:** COVID-19, coronavirus, pandemic, anxiety, COVID-19 anxiety, queue, queueing, queue environment

## Abstract

The COVID-19 pandemic increased global anxiety, and many people shopped less frequently. This study quantifies customer preferences in where to shop while following social distancing regulations, specifically focusing on customers’ anxiety. Collecting data online from 450 UK participants, we measured trait anxiety, COVID-19 anxiety, queue awareness, and queue safety preferences. Confirmatory factor analyses were used to develop novel queue awareness and queue safety preference variables from new items. Path analyses tested the hypothesised relationships between them. Queue awareness and COVID-19 anxiety were positive predictors of queue safety preference, with queue awareness partially mediating the effect of COVID-19 anxiety. These results suggest that customers’ preferences for shopping at one business and not another may depend on safe queueing and waiting conditions, especially in those more anxious about COVID-19 transmission. Interventions that target highly aware customers are suggested. Limitations are acknowledged and areas for future development are outlined.

## 1. Introduction

The COVID-19 pandemic worsened the mental health and well-being of everyday people. During the COVID-19 pandemic, many called for a focus on the mental, not just physical, health issues caused [1,2,3,4]. While there was been an initial emphasis on supporting the mental health of patients and front-line workers [5,6,7], other research since linked trait anxiety and stress to COVID-19 anxiety in general populations [8,9,10]. These authors emphasise the pandemic’s role in causing psychological distress, especially in combination with previously diagnosed anxiety disorders (e.g., Obsessive Compulsive Disorder) [11,12]. A review on young people during the pandemic linked fear of COVID-19 infection to increased anxiety and depressive symptoms such as worrying, irritability, and social isolation [13].

Shopping in-person likely contributed to these increased anxiety levels and worse well-being. During the pandemic, the shopping environment became more hostile, with COVID-19-induced panic buying occurring internationally [14,15]. Sim et al. (2020) theorised that people were panic buying to calm their anxiety by ensuring they and their dependents had enough supplies [16]. Other authors support this conclusion, adding that panic buying was caused by integration of internal feelings such as fear and a need for security, and external factors such as peer influence and government activity [17,18]. As both fear of COVID-19 transmission and having inadequate supplies have been found to be triggers of COVID-19-related stress and anger [19], panic buying and resource scarcity during a pandemic could have further worsened anxiety and well-being while shopping.

Government COVID-19 regulations may not have been enough to make all customers feel secure. Social distancing measures required shops to change their queue systems to limit COVID-19 transmission between customers [20]. For example, some supermarkets made one-way systems which extended queues inside shops [21,22]. However, 80% of consumers in a 2020 survey reported feeling at least somewhat unsafe while away from home [23]. While enforcement of social distancing in queues likely became a basic expectation for consumers, social distancing may not always have been enforced well enough for customers to be comfortable. Even as regulations were reduced, anxious customers may be less likely to go outside generally. Relaxation of some travel restrictions led to overcrowding and neglect of social distancing [24]; similar neglect of social distancing could have also occurred as shopping regulations were reduced too.

Customers changed their spending habits during the pandemic due to anxiety and discomfort. Generally, people reported spending less time shopping, as well as adopting minimalistic spending habits due to their COVID-19 anxiety and social distancing conditions [25,26]. People spent less time in social environments like pubs [27], and even reduced the frequency of their online shopping [28]; customers’ perceived threat of the shopping environment was an influential predictor of these behavioural changes [29]. In a survey early in the pandemic, half of the respondents reported changing their shopping habits due to safety concerns [23]. Additionally, a longitudinal, qualitative study across the COVID-19 pandemic argued that customers generally were first fearful, then frugal as the pandemic continued [30].

The management of customers’ queueing and waiting environments may have contributed to these changes in shopping behaviour. Before the pandemic, queueing literature explored how queues can be enjoyable and increase customers’ perceptions of their waiting environments through managing their attention and anxiety [31,32]. While queueing under pandemic conditions may not ever be enjoyable, good management and regulation of customer queues could still improve customer perceptions of waiting environments. One study during the COVID-19 pandemic showed that people that saw COVID-19 as a bigger threat and people that felt more anxious while queueing showed a preference for more structured waiting environments [33]. When customers have the option of different shops with differently managed queueing and waiting environments, people that are more anxious and threatened by potential infections may have prioritise safety over cost or time efficiency.

**Figure 1:**
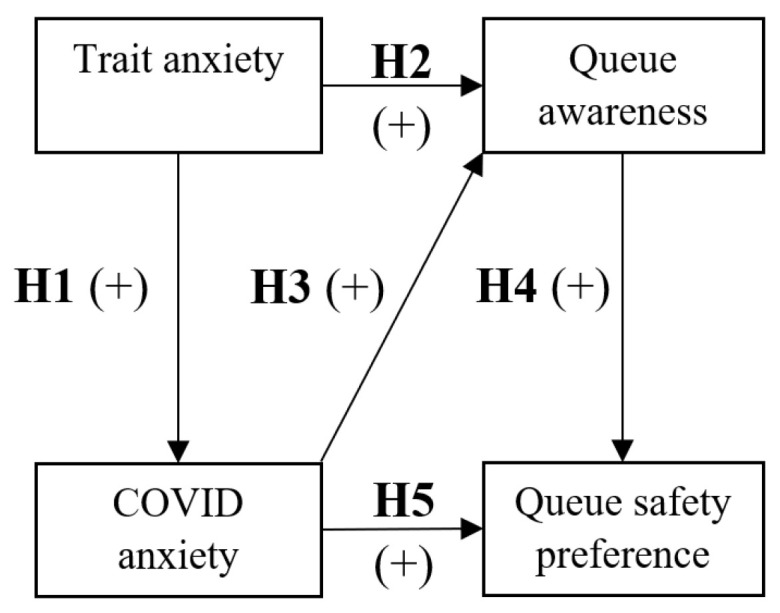
The hypothesised model.

This study looks to quantify the effects that customer anxiety and awareness while queueing had on their preferences for where to shop during the COVID-19 pandemic, asking the question: How does customers’ anxiety while queueing affect their preferences for queue safety during the COVID-19 pandemic? The below theoretical background uses previous pre-pandemic literature to develop a hypothesised model (See Figure 1); this is then tested in our analyses. This study not only looks to develop queueing theory in regard to anxiety, but also links customer experiences within queues to their preferences as to where they shop in-person. While some papers have looked at how other areas of in-person shopping were affected by the COVID-19 pandemic, such as the use of self-service kiosks [34] or even mass panic buying [33], this is the first, to our knowledge, that explores queueing.

## 2. Theoretical Background

### 2.1. COVID-19 Anxiety and Trait Anxiety

Many studies linked the COVID-19 pandemic to increased anxiety and other negative health effects. Anxiety during the pandemic was linked to increased menstrual symptoms [35]; negative emotions and worse academic self-efficacy [36]; and lower sleep quality and less frequent exercise [37]. The number of infected people was also directly linked to anxiety in China [38]. Health, generally, has been described as a dynamic, bidirectional interaction between biological, psychological, and social factors [39]. Therefore, anxiety and these other symptoms could be both the cause and consequence of the decline in international health due to the pandemic.

Anxiety specifically related to COVID-19 has also been researched as an individual difference variable [40]. While the above studies measured individuals’ general state and trait anxiety, COVID-19 anxiety focuses on anxiety specifically related to the pandemic and COVID-19 transmission. When controlling for personality traits and demographics, COVID-19 anxiety has been associated with increased depression and generalised anxiety, accounting for 22.1% and 18.5% unique variance, respectively [12]. These findings strongly suggest that while generalised anxiety and COVID-19 are related, they are two separate constructs. Thus, COVID-19 anxiety may be a separate symptom and cause of declining public health which is distinct from other psychological illnesses. Typically, during pandemic studies, trait and state anxiety and COVID-19 anxiety are measured cross-sectionally (e.g., [12,36]), with COVID-19 anxiety predicting anxiety measures. However, within these studies, it is arguable that this relationship is reversed, with anxiety measures, specifically trait anxiety, predicting levels of COVID-19. Trait anxiety is a characteristic that describes how often people see situations as threatening [41]. Someone with higher trait anxiety would be more vulnerable to anxiety and be more likely to feel anxious in specific situations [42]. During the COVID-19 pandemic, people with higher trait anxiety could be more likely to feel threatened by COVID-19 transmission and its associated health risks, and feel more anxiety in specific circumstances where transmission is possible, such as while queueing. We expect this relationship is considerably stronger than COVID-19 anxiety fundamentally changing how often people respond to threats generally. Therefore, we predict:

**H1:**  *Trait anxiety will be a positive predictor of COVID-19 anxiety*.

### 2.2. Queue Awareness

Prior to the COVID-19 pandemic, queueing or waiting in a more hostile environment could have detrimental impacts on businesses. Liang’s (2019)’s enjoyable queueing model showed that pre-pandemic promotional activities and queue management reduced how aware customers are of the queue and improved their impression of their waiting environment [32]. Historically, studies have used waiting time perceptions as a substitute for enjoyability; if participants perceived that less time passed than actually did, customers were assumed to have had a better experience (e.g., [43,44]; see [45] for a review). However, in Liang’s model, individual differences in waiting and queueing experiences are used to investigate customer experiences in higher resolution [31,32]. These queueing experiences were quantified by items covering anxiety, comfort, unexplained and unspecified waits, group waiting, fair waiting, waiting for a known duration, and the final service value.

During the COVID-19 pandemic, many of these categories were inappropriate for socially distanced queues. Due to the pandemic’s widespread coverage throughout the media, fair, unexplained, and non-specified waiting times were deemed irrelevant; customers would already know about social distancing regulations, and if not, would promptly be informed by staff. Service value was also not included; as we measured queueing conditions generally in this study, it would be hard to determine the quality and quantity of goods people were queueing for. Furthermore, shopping in groups was actively discouraged by supermarkets across the UK [46,47], so group waiting was also not relevant, at least at the time of data collection. Comfort, which could previously refer to seating or sweet scents, may now have a different meaning, especially in queues outside shops. Now, comfort may refer to how at ease customers feel while queueing, which is determined by how aware they are of the queueing environment.

This study measures this queue awareness during the COVID-19 pandemic. The original measurement of queue conditions in Liang’s [31] enjoyable queueing model used a unidimensional model with one item reflecting each of their categories. This study expands on this measurement and proposes a novel, second-order model with the categories relevant under social distancing. The second-order factor, queue awareness, will have two first-order factors: queue anxiety and queue attention. These two factors measure how attentive customers are to the queueing environment, and how threatened they feel while within it. As this study is focused on queues specifically, queue anxiety will function very similarly to state anxiety, but be more specific to the queueing environment. As a result, it is expected that COVID-19 anxiety will predict queue awareness and its factors similarly to how state anxiety has in previous studies (e.g., [12,36]). The next two sections will break down the factors of queue awareness in more detail.

#### 2.2.1. Queue Attention

Waiting theory argues that occupied time feels shorter [48,49]. Much of the literature has investigated how engaging customers even temporarily affects perceived waiting time by distracting their attention from their time spent queueing. For example, while waiting in online queues, visual distractors have increased customer enjoyment [50]. However, this effect has dropped considerably over time, suggesting the effects of these distractions are limited and could expire quickly depending on the amount of content and level of engagement. Recent research has applied this work and created models of enjoyable queueing [31,32]. Here, promotional activities are used to stimulate and engage customers, and together with queue management, indirectly decrease perceived wait time.

Typically, queueing is an established social system with rules and obligations; customers may act cooperatively and according to social pressure [51], and enforce the rules of the queue themselves [52]. While experiments have shown that people allow deviations from these rules [53,54], this tends to be restricted to small cases wherein the loss to other queue members is minimal. Only where queues are less well-defined does the queueing system break down, and customers may become more opportunistic [55]. Under social distancing conditions, queueing systems may be more chaotic, and customers may be more aware of others. While most are cooperative and understanding of social distancing rules, UK citizens have noted that a minority does not conform [56]. As a result, customers may still feel the need to pay attention to others while they are waiting and shopping, regardless of the government’s rules. Without strong, proactive regulation, customers may not trust others to maintain social distancing. Customers may be watching their distance and movement around others; those who would otherwise seek to occupy themselves while waiting and queueing, with their phone or a book, for example [57], may now feel too uncomfortable to do so. Increased attention on the queueing environment works in stark contrast to Liang [32]’s enjoyable queuing model, suggesting that increased queue attention will worsen queueing experiences.

#### 2.2.2. Queue Anxiety

Anxiety is characterised by a sense of uncertainty about something potentially harmful or dangerous [58]. In most cases, it acts as an adaptive defence mechanism, triggering a need to control the situation and choose options that promote survival [59]. This system can be overwhelming and influential on decision making [60]. Generally, anxiety and uncertainty while queueing has been linked to customer dissatisfaction [45,49]. These dissatisfied customers may make different decisions on when, where, and how they shop in the future.

Anxiety interventions have been shown to improve queue and waiting satisfaction. Instead of distracting the customer, anxiety-reducing interventions inform or relax the customer, making them feel more secure while they are waiting. Familiar, ‘likeable’, and fast music has been shown to increase positive emotion in customers [61,62,63,64,65]; pleasant scents, such as vanilla and lavender, have been shown to be most effective in improving customers’ moods when tested in high-anxiety clinical environments [63,66,67], and lower-intensity and softer-coloured light in shades such as blue or green has also shown positive effects on reducing the duration of perceived time [68,69,70]. While there are many ways that anxiety can be reduced, making too many interventions may backfire. Fenko and Loock [66] found that combining music and scent had no effect on relaxing participants. Here, too much environmental stimulation could instead make customers more consciously aware of their waiting environment, preventing any relaxation. While there is overlap between interventions that reduce awareness and anxiety, perhaps the main difference is that anxiety-based interventions are designed to not be paid attention to, while awareness interventions actively draw customers’ attention to them and away from the queueing environment.

Prior to the pandemic, customers preferred to shop when and where they felt safer and more secure. When they feel unsafe, customers have chosen to shop in smaller time windows, in different places, and with different people [71,72]. Businesses have an incentive to prioritise customer safety, as it has been linked to increased customer satisfaction, well-being, and loyalty [73,74,75]. These studies, however, did not measure individual differences in anxiety levels, which could greatly dictate safety preferences. A more anxious customer may appreciate and value business who make pro-safety investments, while less anxious customers may wonder why they are bothering.

Negative experiences within socially distanced queues may cause customers to leave, or shop elsewhere next time. Studies prior to the COVID-19 pandemic have shown that negative queueing experiences have caused individuals to leave queues early, or avoid them altogether [76,77], causing their future shopping choices to change due to the negative emotions they felt [78]. During the COVID-19 pandemic, customers may generally have been experiencing more anxiety in their everyday lives [1,2,3,4], and associating more threat with queueing and waiting environments [33]. As a result, customers may be even more sensitive to poor waiting environments; overwhelmed by anxiety, they may leave unpleasant queues and potentially shop elsewhere to prioritise their safety and well-being.

Therefore, we propose that:

**H2:**  *Trait anxiety will have a positive relationship with queue awareness and its factors.*

**H3:**  *COVID-19 anxiety will have a positive relationship with queue awareness and its factors.*

**H4:**  *Queue awareness will be a positive predictor of queue safety preferences.*

**H5:**  *COVID-19 anxiety will have a positive, indirect effect on customer queue safety preferences through queue awareness.*

## 3. Method

### 3.1. Procedure

Ethics approval was received from an appropriate ethics committee (PSY-S19-016). Participants were recruited during June 2020 through ‘Prolific.ac’, an online participant recruitment website. Data collection ended one day before non-essential shops re-opened after the first UK COVID-19 ‘lockdown’ [79]. Each participant was paid £0.84 and debriefed after the survey. Online sampling was conducted due to its efficiency and due to less risk of COVID-19 transmission. Additionally, online data sampling has shown similar results to in-person sampling [80]. Prolific.ac was used to collect data due to its flexibility in selecting participants and increased sample diversity and data quality compared to its competitors [81,82,83].

### 3.2. Participants

An initial screening survey asked 600 UK residents “Have you participated in more than one queue (for food, pharmaceuticals or otherwise) since social distancing rules have been in place?”. Of the 600, 552 answered affirmatively. These 552 were invited to complete the main survey, of which 491 accepted. Of these cases, 41 were removed due to either failing the attention check or having incomplete data, leaving 450. Some 149 identified as male, and 301 as female. The participants’ mean age was 33.7 years old (SD = 11.70) and most (293; 65.1%) had completed at least an undergraduate degree. The participants’ mean salary was £24,156 (SD = £19,602).

### 3.3. Measures

Trait anxiety was measured by the State-Trait Inventory for Cognitive and Somatic Anxiety (STICSA; [84]). The 21 items measuring cognitive and somatic general mood were used with the original 4-point scale. The STICSA has been shown to have stronger, closer associations with anxiety and weaker relationships with depression measures than other trait anxiety measures [85]. The STICSA has shown good validity and measurement invariance between genders [86,87]. A mean score from all 21 cognitive and somatic items was used in the analysis (composite reliability (CR) = 0.934).

COVID-19 anxiety was measured using a singular item: “How anxious are you about coronavirus (COVID-19) pandemic?”; this was measured on a 7-point scale from “not anxious at all” to “extremely anxious”. This one-item measure has been shown to have adequate validity for data collected at similar times during the pandemic [88,89].

Queue awareness was measured across novel nine items and was also measured on a 7-point scale from “strongly disagree” to “strongly agree”. As described above, items were developed by the authors, one of which is an expert in the area, in accordance with queue and wait-time theory [31,32,45], which was deemed relevant to COVID-19-specific shopping. Reflecting the theoretical background above, queue awareness is a second-order construct, with queue anxiety and queue attention as first-order factors (Final 6-item CR = 0.842).

Queue safety preference was measured across six novel items measured across the same 7-point scale. Items were developed by the authors and focused on customer shopping loyalty decisions that would sacrifice their own resources, such as time and money, for a safer shopping experience (Final 4-item CR = 0.762). For both queue safety preference and queue awareness items (see Table 1 and Table 2), participants were asked to reflect on their specific experiences queueing under social distancing regulations.

Other personality and demographic variables were also measured, but not included in this study.

## 4. Results

### 4.1. Common Method Variance

Common method bias was investigated using Harman’s single factor test [82]. Varimax-rotated generalised least squares estimation was used. The largest factor explained 23.1% of the variance, suggesting the absence of a general factor and minimal common method bias. The factor loadings produced were then used to calculate the average variance explained (0.221), showing similar conclusions.

### 4.2. Confirmatory Factor Analyses

First, confirmatory factor analyses were used to verify the factor structure of novel variables. Multivariate normality was assessed using in R using the *mvn* package [90]. All variables for each had significant multi- and univariate non-normality at *p* < 0.001, as determined by Mardia’s Skewness and Kurtosis tests, and Shapiro–Wilk tests, respectively. Confirmatory factor analyses were run in *R* using the *lavaan* package [91]. Weighted least squares estimation, oblimin rotation, and polychoric correlation matrices were used in calculations to increase accuracy using the non-normal interval data we collected [85,92,93]. Model fit was determined by χ^2^/*df* ratios, comparative fit indices (CFI), Root Mean Square Error of Approximation (RMSEA), and standardised root mean residual (SRMR) scores [94]. Thresholds for excellent fit used were χ^2^/*df* < 3, CFI > 0.95, SRMR < 0.08, RMSEA < 0.05.

#### 4.2.1. Queue Safety Preference

The theorised six-item unidimensional model for queue safety preference met some but not all fit thresholds (χ^2^ = 34.34, *df* = 9, χ^2^/*df* = 3.82, CFI = 0.848, RMSEA = 0.079 (RMSEA 95% CI Lower = 0.052, RMSEA 95% CI Higher = 0.108), SRMR = 0.066). Estimates and modification indices were examined. The item “I would be prepared to spend more for businesses to manage their queues well” was removed from the model due to it having the weakest link to the latent construct. This was also the only item to directly reference financial loss. Additionally, the item “I would be more likely to come back to a business where a queue is effectively managed” had a weak link to the latent variable and notably covaried with “I prefer to shop/bank where I feel safer”. This item was similarly removed. The resultant 4-item final model met all fit statistic thresholds (χ^2^ = 2.468, *df* = 2, χ^2^/*df* = 1.234, CFI = 0.996, RMSEA = 0.023, (RMSEA 95% CI Lower = 0.00, RMSEA 95% CI Higher = 0.099), SRMR = 0.021) and was used in further analysis.

#### 4.2.2. Queue Awareness

The theorised 9-item two-factor second-order queue awareness model did not reach recommended fit thresholds (χ^2^ = 119.261, *df* = 26, χ^2^/*df* = 4.59, CFI = 0.849, RMSEA = 0.089 (RMSEA 95% CI Lower = 0.073, RMSEA 95% CI Higher = 0.106), SRMR = 0.123). In response, queue awareness regression estimates and modification indices were examined. The reversed items “I feel comfortable interacting with other members of the queue”, “I feel other queue members respect my personal space” and “I feel able to relax in queues” all had weaker relationships to the latent constructs and were removed. The resultant 6-item second-order model met all excellent fit thresholds, with the exception of RMSEA, which it only narrowly missed. This model was therefore used in further analysis (See Table 2; χ^2^ = 19.526, *df* = 8, χ^2^/*df* = 2.44, CFI = 0.975, RMSEA = 0.057 (RMSEA 95% CI Lower = 0.025, RMSEA 95% CI Higher = 0.089), SRMR = 0.034).

#### 4.2.3. Trait Anxiety

Finally, the factor structure of the trait anxiety section of the STICSA was assessed. This was carried out across two models, one with a singular trait anxiety as a first-order latent variable, and the second with trait anxiety as a second-order latent variable, with cognitive and somatic anxiety as factors. Both models failed to meet all excellent fit thresholds. The first-order model missed thresholds for χ^2^/*df*, CFI, RMSEA and SRMR (χ^2^ = 657.326, *df* = 189, χ^2^/*df* = 3.48, CFI = 0.583, RMSEA = 0.074 (RMSEA 95% CI Lower = 0.068, RMSEA 95% CI Higher = 0.081), SRMR = 0.240). While the second-factor model had better fit statistics, it still failed to meet the same thresholds (χ^2^ = 595.488, *df* = 187, χ^2^/*df* = 3.18 CFI = 0.901, RMSEA = 0.075 (RMSEA 95% CI Lower = 0.068, RMSEA 95% CI Higher = 0.081), SRMR = 0.049).

As trait anxiety did not meet the fit thresholds, future structural equation modelling investigation uses an average item score for trait anxiety, rather than a calculated latent variable. This allows for clearer interpretation of the fit statistics; any poor fit indices will be due to the relationships in the theoretical model, rather than the trait anxiety STICSA model.

An average trait anxiety variable was chosen over cognitive and somatic anxiety variables to stay in-line with the other measured variables that measure anxiety generally.

### 4.3. Descriptive Statistics

Descriptive statistics of each factor were then explored (See Table 3). All variables were significantly non-normal as determined by Shapiro–Wilk tests (all *p* < 0.001). Therefore, Kendall’s tau coefficients were used. In addition to the model fit statistics, composite reliability and average variance explained values were calculated to assess construct validity [95].

All correlations were significant and positive. Queue anxiety was the largest correlate of both COVID-19 anxiety (*r_t_* = 0.427, *p* < 0.01) and trait anxiety (*r_t_* = 0.249, *p* < 0.01). Queue safety preference was positively correlated with queue attention and queue anxiety (*r_t_* = 0.380, *p* < 0.001 and *r_t_* = 0.314, *p* < 0.01, respectively). COVID-19 anxiety and trait anxiety had amongst the smallest correlations (*r_t_* = 0.193, *p* < 0.01). Queue awareness factors had a moderate but not strong correlation (*r_t_* = 0.371, *p* < 0.001), and had correlations lower than 0.40 with other variables. Cheung and Wang [96] suggest concluding discriminant validity if correlations are not larger than 0.70 between constructs, and concluding convergent validity if AVE and factor loadings are not significantly lower than 0.50. Discriminant validity is reached between constructs, as the highest correlation outside the queue awareness factors is *r_t_* = 0.427. Queue awareness missed the 0.50 threshold, but its factors did not. Therefore, path analyses will be carried out with both the main construct and its factors. While a well-validated measure, trait anxiety also fell below 0.50 AVE. This was also likely due the trait anxiety scale having two factors, of which we also used one in analysis for model parsimony. However, due to its widespread usage of the second-order construct as well as its very high internal reliability, it remained in analysis. Queue safety preference also fell marginally below this 0.50 threshold. This is a measure that should be updated and revised in future study, but it was retained in the analysis due to its excellent model fit statistics and adequate composite reliability.

### 4.4. Structural Equation Modelling and Path Analyses

First, structural equation modelling was used to test the hypothesised model, with queue awareness and queue safety preference as latent variables; queue awareness was used as a second-order factor with the model’s hypothesised relationships including this latent variable rather than its subfactors. Structural equation modelling used the same settings as the above confirmatory factor analyses. This model failed to meet all fit statistic thresholds, showing moderate deviations in each case (χ^2^ = 171.582, *df* = 49, χ^2^/*df* = 3.50, CFI = 0.825, RMSEA = 0.075 (RMSEA 95% CI Lower = 0.063, RMSEA 95% CI Higher = 0.081), SRMR = 0.099). Inspection of the modification indices suggested that a lack of covariances between the latent variables, particularly between queue attention and anxiety and queue safety preferences could contribute to the poor model fit.

To attempt to bypass this issue, a second structural equation model was run without queue awareness as a second-order variable. Queue anxiety and queue attention factors were set as their own latent variables, and these two latent variables were allowed to covary. This simplifies the model, and removes the need for additional covariances between latent variables. This model fit two of the four excellent fit statistic thresholds, one ‘goodness of fit’ statistic, χ^2^/*df* < 3, and one ‘badness of fit’ statistic, SRMR < 0.08 (χ^2^ = 136.610, *df* = 47, χ^2^/*df* = 2.91, CFI = 0.875, RMSEA = 0.065 (RMSEA 95% CI Lower = 0.053, RMSEA 95% CI Higher = 0.078), SRMR = 0.071). RMSEA scores were close to the threshold, while CFI scores deviated more. Inspection of the modification indices showed that these smaller deviations would be improved by adding covariances between measured items for the latent variables, and addition to between COVID-19 and a couple of the measured items. While this model is arguably passable, especially when adding these covariances in, we decided to use a path analysis with the measured variables to simplify the model and the mediation analysis. While this reduces the accuracy of the latent variable measurement, it means that deviations from model fit will be due to the relationships in the hypothesised model, rather than due to a lack of covariances between measured items.

**Figure 2:**
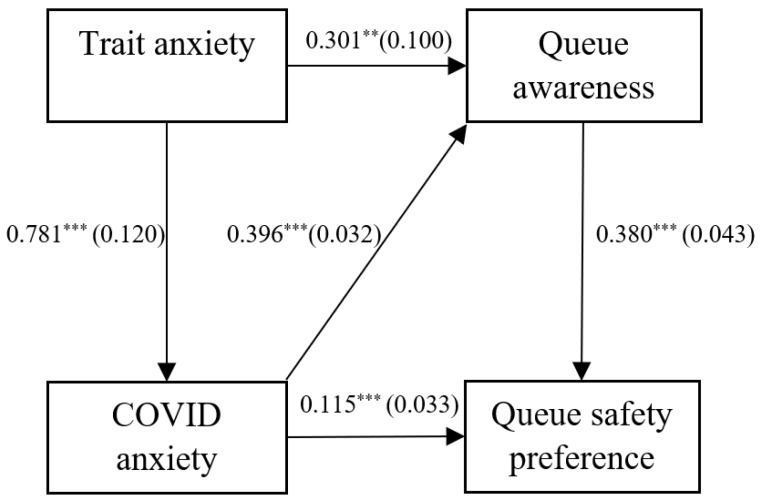
Final path analysis using queue awareness as an observed variable. Unstandardised estimates are shown between variables with standard errors in parentheses. ** *p* < 0.01, *** *p* < 0.001.

The hypothesised model shown in Figure 1 was then tested using path analysis. Path analyses were calculated using R’s *lavaan* package [91] to facilitate the mediation and moderation analysis in the model. As this used mean variables scores as opposed to Likert-scale data, robust maximum likelihood estimation was used in calculation. This model had excellent fit, meeting all thresholds (χ^2^ = 1.081, df = 1, χ^2^/*df* = 1.081, CFI = 1, RMSEA = 0.013, (RMSEA 95% CI Lower = 0.000, RMSEA 95% CI Higher = 0.127), SRMR = 0.012; see Figure 2 and Table 4). All relationships were significant and in accordance with our hypotheses.

**Figure 3:**
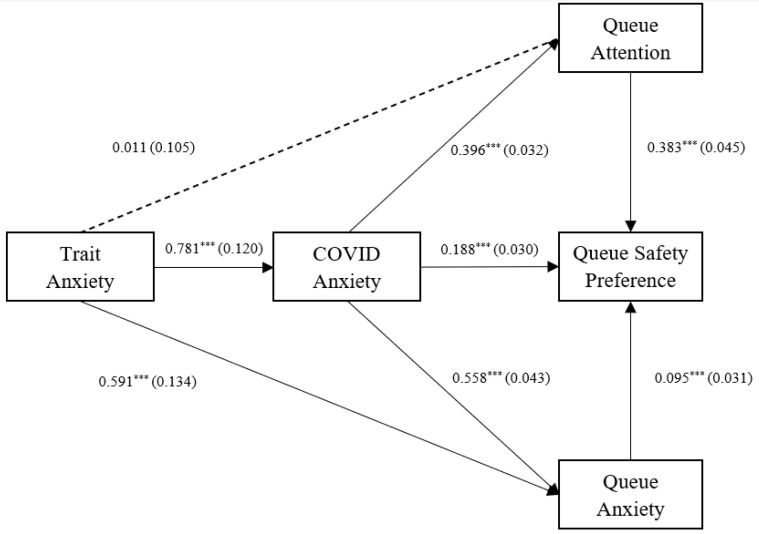
Final path analysis using queue attention and queue anxiety as observed variables. Unstandardised estimates are shown between variables with standard errors in parentheses. *** *p* < 0.001.

A similar model was then analysed to investigate the individual relationships of the queue awareness factors, queue anxiety and queue attention. This resultant model, as shown in Figure 3, met all excellent fit thresholds (χ^2^ = 0.087, df = 1, χ^2^/*df* = 0.87, CFI = 1, RMSEA = 0.000 (RMSEA 95% CI Lower = 0.000, RMSEA 95% CI Higher = 0.084), SRMR = 0.003; see Figure 3 and Table 5). In this model, trait anxiety was a positive predictor of queue anxiety, but not queue attention. Both queue attention and queue anxiety were significant, unique, positive predictors of queue safety preference. queue attention was a stronger predictor than queue anxiety. COVID-19 anxiety had a significant positive, indirect effect on queue safety preference through both queue attention and queue anxiety, while also having its own direct effect.

Gender differences in the model were then explored by testing for measurement invariance between participating men and women. Measurement invariance testing was again carried out using R’s *lavaan* package (Rosseel, 2012), which tests for the same factor structure (configural variance), the same factor loadings (weak invariance) and the same factor loadings and intercepts (strong variance across groups) using the scaled Chi-squared different test. Testing showed that for both queue awareness first and second-order factor models above, there were no significant differences in factor structure, loadings, or intercepts between gender-specific models (*p* ≥ 0.06).

## 5. Discussion

This study expanded on the measurement of queue awareness and explored its factor structure. It also tested our hypothesised model, measuring how anxiety while queueing affects customers preferences for safety, as well as assessing the mediating effect of queue awareness between COVID-19 anxiety and queue safety preferences.

Trait anxiety was a positive predictor of COVID-19 anxiety, supporting **H1** and prior research [12,40]. Notably, however, the correlation between the two variables was small and explained less variance than these previous studies. As our data were collected later in the pandemic, this could suggest that the relationship between trait anxiety and COVID-19 anxiety is diminishing over time, perhaps as contextual factors such as local case or death rates become more influential than personality traits. Alternatively, this difference could be explained by the different methods of COVID-19 anxiety measurement. This study uses a one-item general measure, while Lee [40] and Lee et al. [12] used multiple items measuring COVID-19 anxiety in specific areas. Furthermore, our decision to analyse trait anxiety as an individual variable, rather than as two variables of cognitive and somatic anxiety, may have obscured any relationship between the variables. This could have led to trait anxiety’s higher standard error scores in the path analysis models.

Trait anxiety and COVID-19 anxiety were positive predictors of queue awareness, supporting **H2** and **H3**. Specifically, trait anxiety was a positive predictor of queue anxiety, not queue attention, when controlling for COVID-19 anxiety. This reflects previous research that shows poor queueing environments are anxiety-inducing and uncomfortable [45]. Customers feeling more uneasy has previously been linked to worse queueing and waiting experiences [31,32], and our research has reflected this. Anxiety over the risk of COVID-19 transmission could therefore be drawing people’s attention away from attempts to occupy themselves, and causing them to become more aware of the positions of others.

Furthermore, COVID-19 anxiety and queue awareness were both significant positive predictors of queue safety preference, supporting **H4**; queue awareness also partially mediated the effect of COVID-19 anxiety on queue safety preference, supporting **H5**. Of the two queue awareness factors, queue attention was a considerably larger predictor of queue safety preference. This is supported by previous interview studies on customer behaviour at shopping centres prior to the pandemic [71,72] and is also in-line with other quantitative research that show links between customer safety, satisfaction, well-being, and loyalty [73,74,75].

### 5.1. Theoretical Implications

This study found evidence for a factor-based structure of queue awareness and revealed its factors had slightly different effects. Previously, Liang [31,32] had used only a unidimensional measurement to measure customer perceptions while queueing. Instead, we used a novel 6-item, two-factor queue awareness model of queue anxiety and queue attention. These factors were shown to have marginally different relationships with the other variables in the study. For example, after controlling for COVID-19 anxiety in the mediation analysis, trait anxiety was a significant, positive predictor of queue anxiety, but not queue attention. Naturally, these factors may have unique relationships with other variables too, differing in direction and magnitude. A factor approach to individual differences in customer experience while queueing and/or waiting may highlight specific relationships between them and customer behaviour.

However, the second-order factor model presented in this study is by no means complete. Due to social distancing regulations, many parts of queue awareness, such as queue socialisation, group, and unexplained waiting, were deemed irrelevant. Naturally, however, in queues without social distancing, these all may contribute to queue awareness and interact differently with other subfactors and measures. Further development and more extensive validation are needed.

Our results show that people more anxious and aware of the queue environment prefer to shop in safer shops during socially distanced conditions. As a result, appropriate management of queue systems may motivate customers to visit and return to businesses, even if they are more anxious about transmission. Additionally, more anxious customers may identify with, and feel more emotionally attached to businesses that manage their queueing systems, and this has been seen previously [97,98]. This study also supports other COVID-19-themed studies’ applications of protection motivation theory, adding to the quantitative evidence showing that consumers’ spending and shopping habits are affected by negative mood, whether that be through perceived threat, vulnerability or anxiety [29,99].

### 5.2. Practical Implications

Managing socially distanced queues effectively may help businesses as well as customers to stay resilient during pandemics. Previous research has argued that managing customer feelings of community and relatedness may help a business to survive through the COVID-19 pandemic [100]. While this was originally proposed for the tourism industry, our results suggest that this could be more generally applicable, increasing customers’ preferences to shop at specific businesses.

Primarily, our results show the importance of queue attention on customer preferences. Queue attention, in addition to queue safety preference, had the highest mean scores of the variables measured on a 7-point scale, 5.44 and 5.49, respectively. Furthermore, queue attention had the largest individual effect on queue safety preference. As COVID-19 anxiety may be difficult to manage as a business alone, queue attention could be both the best and most practical target to reduce customers’ anxiety and encourage them to shop during the pandemic and support businesses. Therefore, businesses that enforce customer movement more strictly, in queues inside and outside, may see more new and returning customers. Additionally, businesses could offer alternatives to in-person shopping in high-anxiety circumstances. One example of this could be through developing or using delivery services. This would allow more anxious customers to purchase enough food while minimising the risk of COVID-19 exposure. Some international studies have linked threat-based variables to a preference for online shopping, with variables of perceived fear [101] and perceived severity of the COVID-19 pandemic [102] (see Li et al., [103] for a review). Theoretically too, these threatening, anxiety-inducing conditions could encourage people who otherwise would not to start using online delivery applications [104]. This study, alongside these articles, may explain the soar in the use of online UK food delivery services during the COVID-19 pandemic (e.g., [105]).

### 5.3. Limitations and Future Research

Firstly, this research is limited by its design; the data is cross-sectional. While causality is assumed in the path analyses, it cannot be inferred from data collected simultaneously. For example, queue anxiety and queue attention could likely both have reciprocal relationships with COVID-19 anxiety. Noticing other customers’ non-compliance to social distancing norms may increase queue attention and queue anxiety; similarly, high compliance may soothe customers’ COVID-19 anxiety.

Additionally, the variables are limited by their size and validation. While there were good composite reliability and excellent fit statistics for each of the novel models analysed, average variance explained scores did not always meet the fit thresholds. For example, queue safety preference fell under the >0.50 suggestions, with a score of 0.451. This could be due to some of its items being less explicitly queue-related, such as #2 and #3, despite instructions for participants to reflect upon their time within queues. In addition, factors were only calculated from 3–4 items. This study did suggest that these variables have concurrent validity, through their relationships with trait and COVID-19 anxiety; however, the issues with the novel variables could have contributed to the sub-excellent structural equation model fit statistics, leading to the analysis using path analyses instead. Further exploration of queue awareness during both socially distanced and normal queue environments could further develop its measurement. The ecological validity is of this study is also limited. Customers were not actively in queues while answering the questions, as it would be impractical under social distancing guidelines at the time of collection. Instead, they were instructed to answer questions on their memory of queueing under the new guidelines. Furthermore, in measuring queue safety preferences and not observing consumer behaviour, our conclusions are distorted, putting heavy emphasis on how customers feel, rather than what they do. Normally, consumer behaviour would integrate both their feelings and practical considerations (e.g., pricing and time). This interaction, however, is not measured; as such, real shopping behaviour while social distancing is difficult to estimate.

The timing of our data collection is an important consideration. These data were collected at the end of the first UK lockdown (June 2020), when COVID-19 was still new to people and only supermarkets, pharmacies, and banks were permitted to open. Months later, over several lockdowns, attitudes towards social distancing may have changed. Other industries have also been permitted to open, at least for some of the time. Future research is needed to fully explore how customer attitudes and anxieties may have changed over time, and even which demographics are most liable to change.

These results suggest that interventions may have varied effectiveness between customers. Our study specifically focuses on individual differences in queue awareness and queue safety preferences, and shows their variation. What is unacceptable to one customer may be very comfortable for another. Early in the COVID-19 pandemic, some UK supermarkets opened exclusively for vulnerable groups, such as the elderly, at scheduled times during the week [106]. Again, supermarkets could benefit from such restrictions. Future research could investigate the effectiveness of booking systems for shops and supermarkets for some opening hours. Customers with high queue awareness may be more motivated to attend these at these times if they are ensured that the number of other customers will be limited. Perhaps less intrusively, studies could also investigate how publicising customer numbers at different times of day affects customer numbers and awareness while queueing. This information would allow those more attentive to queueing and waiting conditions to plan their shopping for a time at which there may be less risk of transmission.

## 6. Conclusions

This study tests a model of how anxiety relates to queue awareness and customers safety preferences on where to shop. Our results show positive relationships between these variables, with the queue attention factor of queue awareness being the single largest contributor to queue safety preference scores. Interventions in supermarkets could be developed to help their more anxious, aware, and attentive customers, and to maximise their well-being while having minimal impact on other customers that are less negatively affected by anxiety over COVID-19 transmission. This study also attempts to quantify queue awareness as a second-order construct. Due to social distancing conditions, this model is inherently incomplete, as other factors previously found to affect the queueing experience are less relevant. The authors heavily encourage further and more extensive development of queue awareness measurement in socially distanced and normal queueing conditions.

## Figures and Tables

**Table 1:** Descriptive statistics and estimates from the queue safety preference confirmatory factor analysis.

Queue Safety Preference	M	SD	Model 1 (6-Item)				Model 2 (4-Item)			
			Estimate	*SE*	*z*	*p*	Estimate	*SE*	*z*	*p*
1. I would be prepared to spend more for businesses to manage their queues well	3.86	1.656	0.573	0.085	6.751	0.000				
2. I prefer to shop/bank where I feel safer	5.58	1.185	0.783	0.051	15.329	0.000	0.843	0.056	15.118	0.000
3. Getting food and supplies safely is my top priority	5.51	1.239	0.939	0.064	14.775	0.000	1.012	0.071	14.259	0.000
4. I would be happy to spend more time waiting in a queue where I know other customers will stay outside my personal space	5.24	1.450	0.891	0.063	14.209	0.000	0.794	0.069	11.520	0.000
5. I would be more likely to come back to a business where a queue is effectively managed	5.93	1.097	0.767	0.066	11.576	0.000				
6. I would not use businesses that I knew had poorly managed queues	5.29	1.509	0.995	0.067	14.767	0.000	0.831	0.085	9.806	0.000

For both models, the variance of the latent variable was constrained to 1 instead of the first observed variable.

**Table 2:** Descriptive statistics and estimates from the queue awareness confirmatory factor analysis.

			Model 1 (9-Item)				Model 2 (6-Item)			
Item	M	SD	Estimate	*SE*	*z*	*p*	Estimate	*SE*	*z*	*p*
I feel alert when I am queueing	5.25	1.408	1.00				1.000			
I am more conscious of the time I spend in the queue	5.40	1.432	0.985	0.104	9.464	0.000	0.894	0.095	9.434	0.000
I am more aware of my surroundings when I am queueing	5.67	1.264	0.895	0.083	10.765	0.000	1.015	0.092	11.079	0.000
I feel comfortable interacting with other members of the queue	3.44	1.734	−1.117	0.142	−7.784	0.000				
I feel other queue members respect my personal space	4.29	1.586	−1.049	0.134	−7.855	0.000				
I feel vulnerable in queues	3.94	1.840	1.000				1.000			
I am concerned for my health while queueing	4.02	1.837	1.008	0.030	34.169	0.000	0.952	0.034	28.252	0.000
I feel anxious in queues	3.97	1.923	1.072	0.034	31.858	0.000	0.957	0.038	24.870	0.000
I feel able to relax in queues	3.54	1.593	−0.706	0.039	−18.015	0.000				

**Table 3:** Descriptive statistics, composite reliability, Kendall’s tau correlation coefficients.

	M	SD	CR	AVE	1	2	3	4	5	6
1. COVID anxiety	4.23	1.54								
2. Trait anxiety	1.76	0.559	0.934	0.410	0.193 **					
3. Queue safety preference	5.49	1.02	0.762	0.451	0.326 **	0.083 *				
4. Queue attention	5.44	1.11	0.757	0.516	0.256 **	0.100 **	0.380 **			
5. Queue anxiety	3.98	1.70	0.898	0.746	0.427 **	0.249 **	0.314 **	0.371 **		
6. Queue awareness	4.71	1.22	0.842	0.487	0.410 **	0.210 **	0.379 **	0.616 **	0.800 **	

CR = composite reliability, AVE = average variance explained; * *p* < 0.05, ** *p* < 0.01.

**Table 4:** Queue awareness path analysis.

Predictor	Outcome	R^2^	Estimate	*SE*	*Z*	*p*
Trait anxiety	COVID anxiety	0.081	0.781	0.120	6.515	0.000
Trait anxiety	Queue awareness	0.305	0.301	0.100	2.995	0.0003
COVID anxiety	Queue awareness (a)		0.396	0.032	12.468	0.000
COVID anxiety	Queue safety preference (c)	0.326	0.115	0.033	3.481	0.000
Queue awareness	Queue safety preference (b)		0.380	0.043	8.755	0.000
Indirect Effect (a × b)			0.150	0.023	6.648	0.000
Total effect (c + (a × b))			0.266	0.032	8.399	0.000

**Table 5:** Queue anxiety and attention path analysis.

Predictor	Outcome	R^2^	Estimate	*SE*	*z*	*p*	*Std.all*
Trait anxiety	COVID anxiety	0.081	0.781	0.120	6.515	0.000	0.284
Trait Anxiety	Queue anxiety	0.345	0.591	0.134	4.393	0.000	0.194
COVID anxiety	Queue anxiety (a1)		0.558	0.043	12.991	0.000	0.502
Trait Anxiety	Queue attention	0.104	0.011	0.105	0.103	0.918	0.005
COVID anxiety	Queue attention (a2)		0.234	0.036	6.506	0.000	0.321
COVID anxiety	Queue safety preference (c)	0.351	0.140	0.034	4.164	0.000	0.140
Queue anxiety	Queue safety preference (b1)		0.095	0.031	3.023	0.002	0.159
Queue attention	Queue safety preference (b2)		0.335	0.048	6.989	0.000	0.369
Indirect Effect 1 (a1 × b1)			0.053	0.018	2.934	0.003	0.080
Indirect Effect 2 (a2 × b2)			0.078	0.017	4.597	0.000	0.119
Total effect (c + (a1 × b1) + (a2 × b2))			0.272	0.032	8.509	0.000	0.212
Queue Anxiety and Queue Attention Covariance			0.565	0.081	6.981	0.000	0.387

## Data Availability

Data is available from the authors upon request.
